# Supplementary material for: Elucidation of host and symbiont contributions to peptidoglycan metabolism based on comparative genomics of eight aphid subfamilies and their Buchnera
Source: PLoS Genet. 2022 May 6;18(5):e1010195. doi: 10.1371/journal.pgen.1010195 (PMC9116674; doi:10.1371/journal.pgen.1010195)
Supplement: S2 Table — (DOCX) [file pgen.1010195.s002.docx]

**S2 Table**

| Species | Number of raw read pairs  (Paired-end) | Number of filtered read pairs (Paired-end) | Number of filtered reads (Single-end) | | Total clean data |
| --- | --- | --- | --- | --- | --- |
|  |  |  | 1.fq | 2.fq | Paired + Single |
| *Geopemphigus sp.* | 24,208,694 | 22,990,388 | 1,112,376 | 70,667 | 7G |
| *Stegophylla sp.* | 132,926,971 | 129,705,617 | 2,127,251 | 849,844 | 38G |
| *Pemphigus obesinymphae* | 174,557,402 | 172,335,182 | 1,413,123 | 648,179 | 51G |
| *Chaitophorus viminalis* | 197,557,815 | 195,096,160 | 1,522,203 | 713,029 | 58G |
