# Supplementary material for: Elucidation of host and symbiont contributions to peptidoglycan metabolism based on comparative genomics of eight aphid subfamilies and their Buchnera
Source: PLoS Genet. 2022 May 6;18(5):e1010195. doi: 10.1371/journal.pgen.1010195 (PMC9116674; doi:10.1371/journal.pgen.1010195)
Supplement: S3 Table — (DOCX) [file pgen.1010195.s003.docx]

**S3 Table**

| Species | Number of raw read pairs (Paired-ended) | Number of filtered read pairs (Paired-ended) | Number of filtered reads (Single-ended) | | Total clean data |
| --- | --- | --- | --- | --- | --- |
|  |  |  | 1.fq | 2.fq |  |
| *Geopemphigus sp.* | 19,151,527 | 18,798,683 | 200,306 | 120,174 | 6G |
| *Stegophylla sp.* | 18,781,778 | 18,446,558 | 194,193 | 112,375 | 5G |
| *Pemphigus obesinymphae* | 22,695,559 | 22,558,652 | 81,582 | 46,859 | 7G |
| *Chaitophorus viminalis* | 21,402,531 | 21,281,141 | 67,402 | 47,981 | 6G |
