# Supplementary material for: Elucidation of host and symbiont contributions to peptidoglycan metabolism based on comparative genomics of eight aphid subfamilies and their Buchnera
Source: PLoS Genet. 2022 May 6;18(5):e1010195. doi: 10.1371/journal.pgen.1010195 (PMC9116674; doi:10.1371/journal.pgen.1010195)
Supplement: S4 Table — (DOCX) [file pgen.1010195.s004.docx]

**S4 Table**

| Species | Total length | N50 | N90 | Maximum scaffold length |
| --- | --- | --- | --- | --- |
| *Geopemphigus sp.* | 244,875,386 | 41,816 | 5,926 | 267,905 |
| *Stegophylla sp.* | 635,813,851 | 10,855 | 332 | 163,545 |
| *Pemphigus obesinymphae* | 658,653,352 | 43,539 | 4,495 | 390,078 |
| *Chaitophorus viminalis* | 437,832,569 | 119,959 | 439 | 1,086,150 |
