# Supplementary material for: Elucidation of host and symbiont contributions to peptidoglycan metabolism based on comparative genomics of eight aphid subfamilies and their Buchnera
Source: PLoS Genet. 2022 May 6;18(5):e1010195. doi: 10.1371/journal.pgen.1010195 (PMC9116674; doi:10.1371/journal.pgen.1010195)
Supplement: S5 Table — (DOCX) [file pgen.1010195.s005.docx]

**S5 Table**

| Species | Complete | Complete and single-copy | Complete and duplicated | Fragmented | Missing |
| --- | --- | --- | --- | --- | --- |
| *Geopemphigus sp.* | 99.2% | 98.7% | 0.5% | 0.4% | 0.4% |
| *Stegophylla sp.* | 97.7% | 95.9% | 1.8% | 1.4% | 0.9% |
| *Pemphigus obesinymphae* | 99.3% | 97.6% | 1.7% | 0.2% | 0.5% |
| *Chaitophorus viminalis* | 97.2% | 95.9% | 1.3% | 0.4% | 2.4% |
