# Supplementary material for: Elucidation of host and symbiont contributions to peptidoglycan metabolism based on comparative genomics of eight aphid subfamilies and their Buchnera
Source: PLoS Genet. 2022 May 6;18(5):e1010195. doi: 10.1371/journal.pgen.1010195 (PMC9116674; doi:10.1371/journal.pgen.1010195)
Supplement: S6 Table — (DOCX) [file pgen.1010195.s006.docx]

**S6 Table**

| Species | Complete | Complete and single-copy | Complete and duplicated | Fragmented | Missing |
| --- | --- | --- | --- | --- | --- |
| *Geopemphigus sp.* | 98.8% | 98.0% | 0.8% | 0.6% | 0.6% |
| *Stegophylla sp.* | 97.1% | 94.5% | 2.6% | 1.6% | 1.3% |
| *Pemphigus obesinymphae* | 98.6% | 96.5% | 2.1% | 0.8% | 0.6% |
| *Chaitophorus viminalis* | 97.4% | 95.4% | 2.0% | 0.4% | 2.2% |
