# Supplementary material for: Elucidation of host and symbiont contributions to peptidoglycan metabolism based on comparative genomics of eight aphid subfamilies and their Buchnera
Source: PLoS Genet. 2022 May 6;18(5):e1010195. doi: 10.1371/journal.pgen.1010195 (PMC9116674; doi:10.1371/journal.pgen.1010195)
Supplement: S10 Table — (DOCX) [file pgen.1010195.s010.docx]

**S10 Table**

| Probe | Sequence (5’-3’) |
| --- | --- |
| Cy5-ApisP2A | (Cy5)-CCAACAAGCTAATCTCGTCT |
| Cy3-PASSisR | (Cy3)-CCCGACTTTATCGCTGGC |
