# Supplementary material for: Elucidation of host and symbiont contributions to peptidoglycan metabolism based on comparative genomics of eight aphid subfamilies and their Buchnera
Source: PLoS Genet. 2022 May 6;18(5):e1010195. doi: 10.1371/journal.pgen.1010195 (PMC9116674; doi:10.1371/journal.pgen.1010195)
Supplement: S12 Table — Tm values were calculated for the specific DNA polymerase used for each primer set. Sequence in bold denotes restriction enzyme recognition sites, while underlined sequence highlights the target sequence used to calculate the Tm. (DOCX) [file pgen.1010195.s012.docx]

**S12 Table**

| Primer | Description | Tm (˚C) | Sequence (5’-3’) |
| --- | --- | --- | --- |
| 1 | *Ec*AmiD-fwd | 63 | CGCGGCAGC**CATATG**GCAGGCGAAAAAGGCATTGTC |
| 2 | *Ec*AmiD-rvs | 65 | GTGGTGGTG**CTCGAG**CTAATCCTGCCCGTATTTCTCCAG |
| 3 | T7 | 48 | TAATACGACTCACTATAGGG |
| 4 | T7term | 55 | GCTAGTTATTGCTCAGCGG |
