# Supplementary figures and images for: Elucidation of host and symbiont contributions to peptidoglycan metabolism based on comparative genomics of eight aphid subfamilies and their Buchnera
Source: PLoS Genet. 2022 May 6;18(5):e1010195. doi: 10.1371/journal.pgen.1010195 (PMC9116674; doi:10.1371/journal.pgen.1010195)

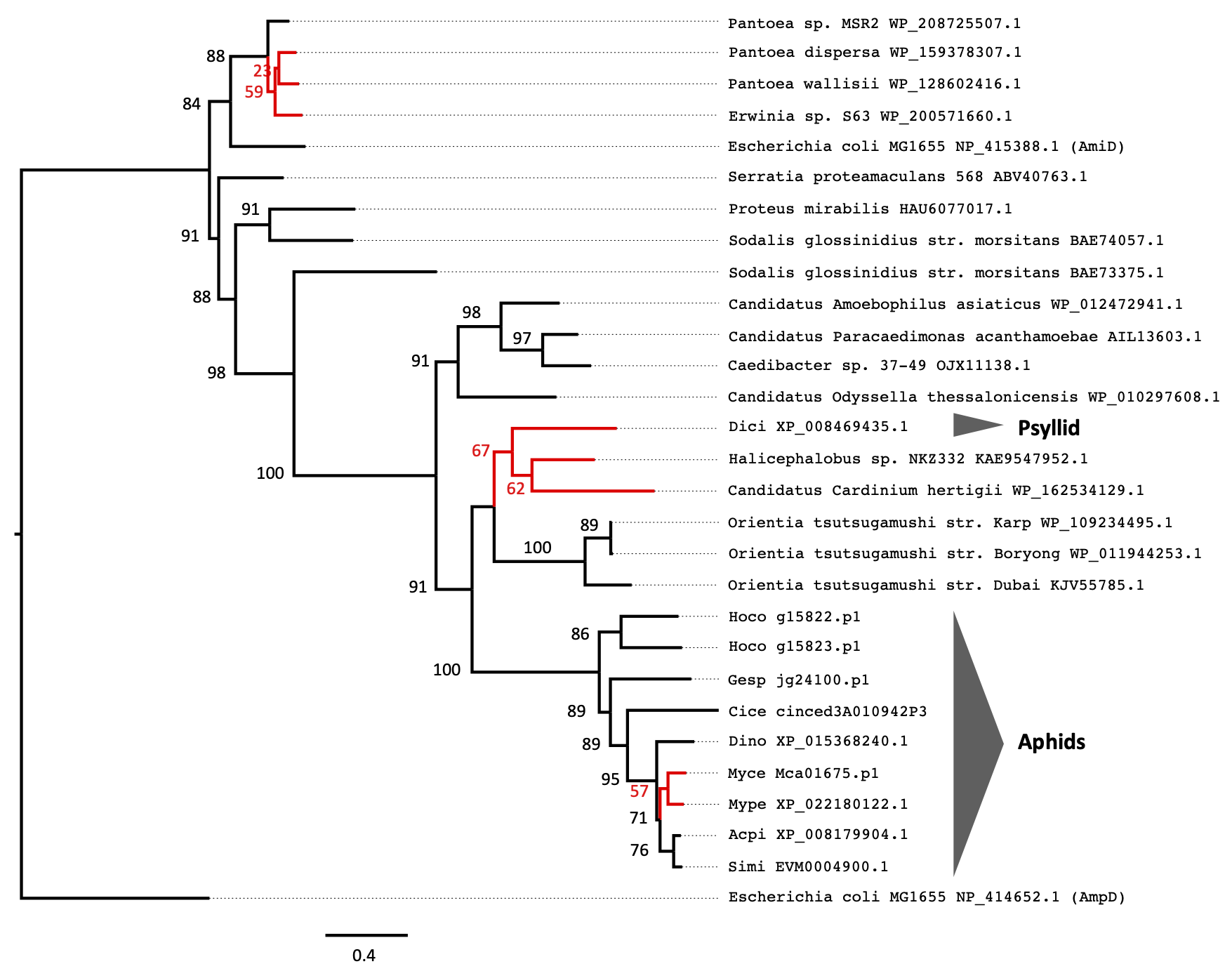

Supplement: S1 Fig — An alignment of 256 amino acid residues was used to construct a ML tree with 100 bootstraps. TBE was used to determine bootstrap supports (88). Branches with <70% support are indicated in red. The scale bar indicates the number of substitutions per site. The tree was rooted using the E. coli AmpD amidase protein. (TIF) [file pgen.1010195.s013.tif]

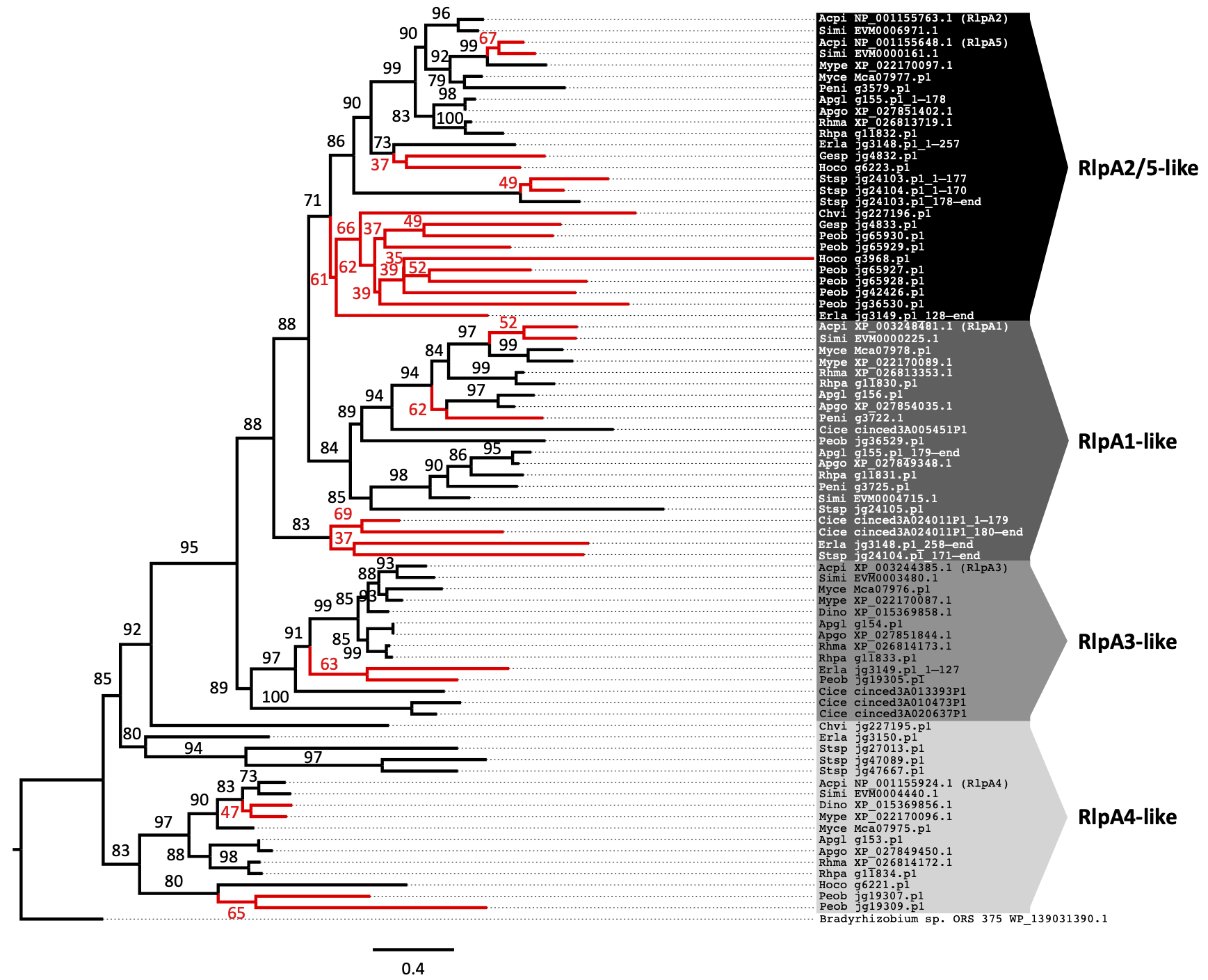

Supplement: S2 Fig — An alignment of 160 amino acid residues was used to construct a ML tree with 100 bootstraps. TBE was used to determine bootstrap supports (88). Branches with <70% support are indicated in red. The scale bar indicates the number of substitutions per site. The tree was rooted using the closest BLAST hit for the A. pisum RlpA4 protein, as phylogenetic positioning of the A. pisum RlpA proteins has shown that identity of the bacterial source of aphid rlpA is unclear (13). Some distinct RlpA sequences were predicted to be derived from a single coding sequence—in these instances, the amino acid range is specified. (TIF) [file pgen.1010195.s014.tif]

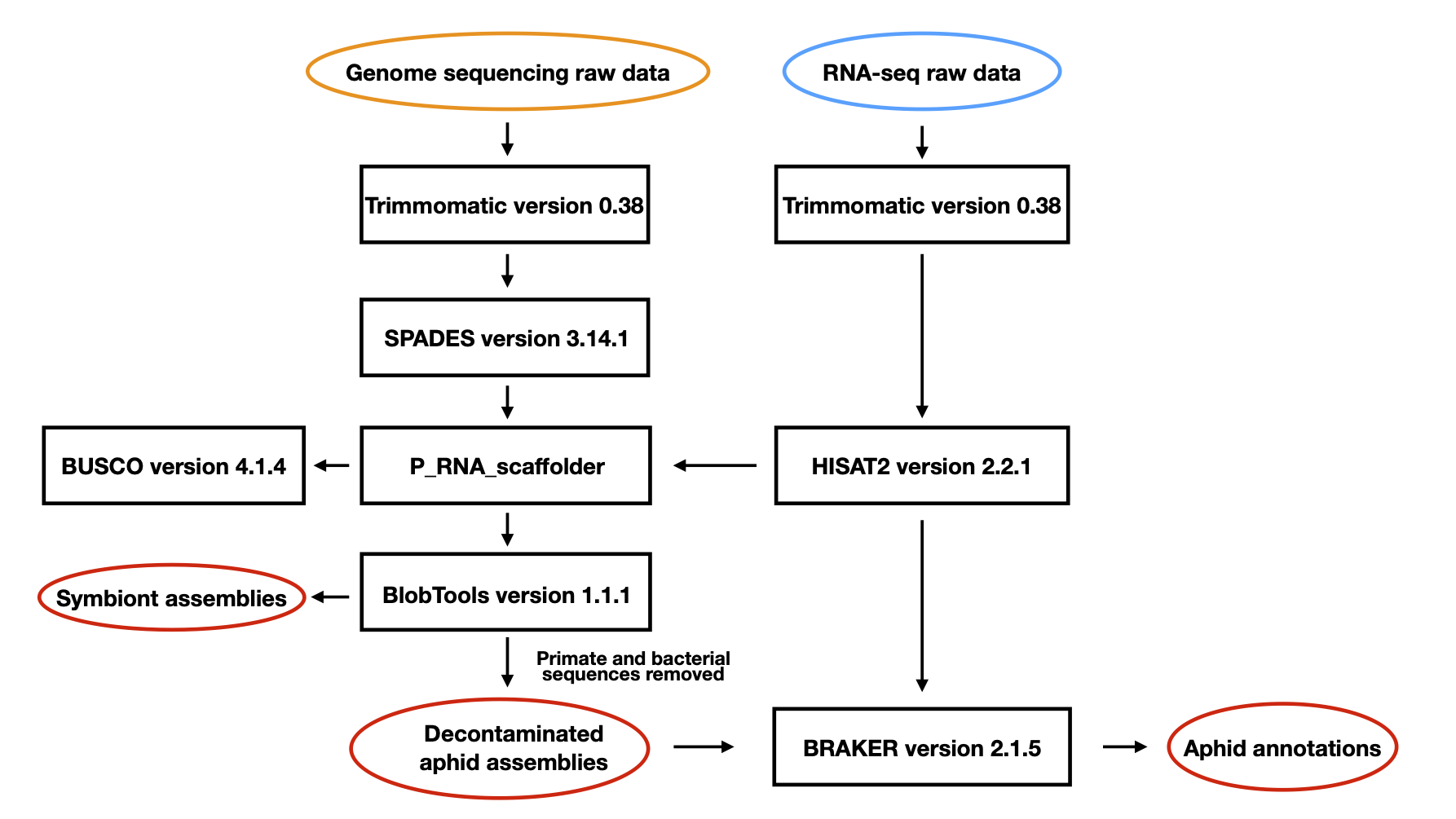

Supplement: S3 Fig — (TIF) [file pgen.1010195.s015.tif]

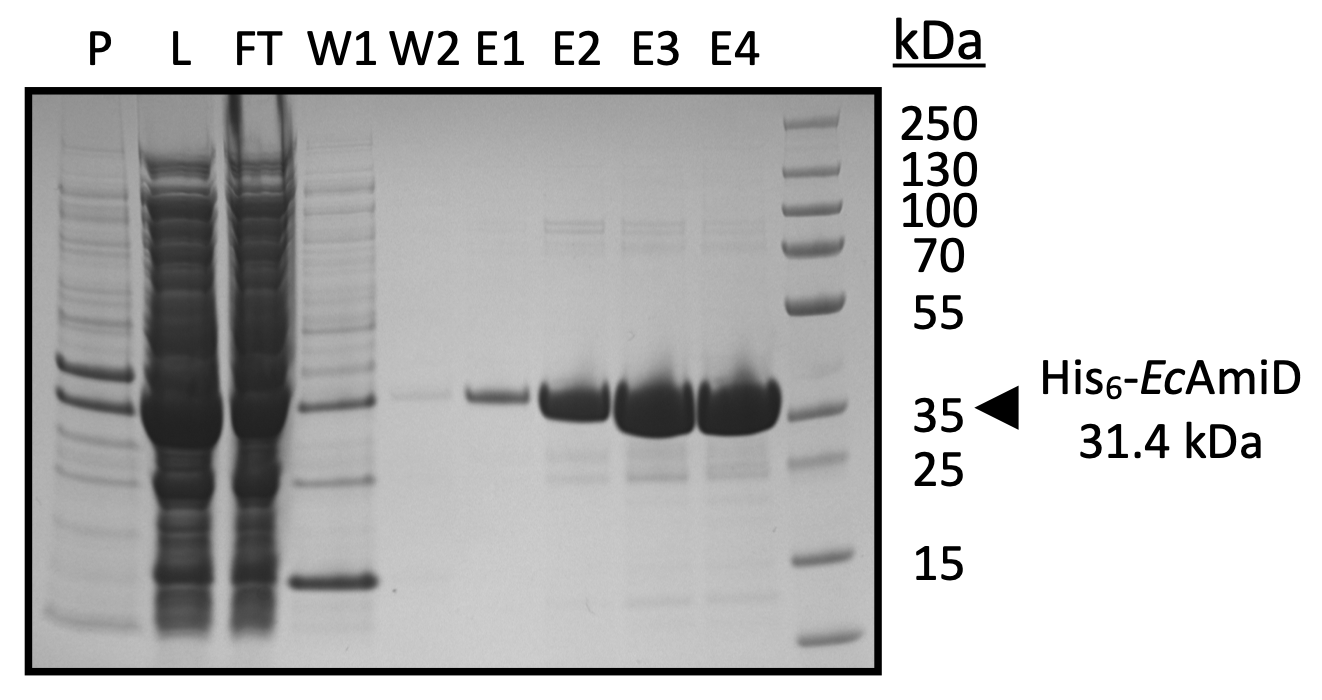

Supplement: S4 Fig — Fractions analyzed include the insoluble pellet (P), soluble cell lysate (L), Ni-NTA flow-through (FT), lysis buffer wash (W1), HEPES buffer wash (W2), and HEPES-buffered elutions of increasing imidazole concentration: 50 mM (E1), 100 mM (E2), 200 mM (E3), and 500 mM (E4). (TIF) [file pgen.1010195.s016.tif]
