# Supplementary material for: Elucidation of host and symbiont contributions to peptidoglycan metabolism based on comparative genomics of eight aphid subfamilies and their Buchnera
Source: PLoS Genet. 2022 May 6;18(5):e1010195. doi: 10.1371/journal.pgen.1010195 (PMC9116674; doi:10.1371/journal.pgen.1010195)
Supplement: Attachment — Submitted filename: v2.2_review_comments_v2.docx [file pgen.1010195.s017.docx]

Editor comments:

The manuscript was fully evaluated at the editorial level and by independent peer reviewers. The reviewers appreciated the attention to an important problem, but raised some substantial concerns about the current manuscript. Based on the reviews, we will not be able to accept this version of the manuscript, but we would be willing to review a much-revised version. We cannot, of course, promise publication at that time.

Should you decide to revise the manuscript for further consideration here, your revisions should address the specific points made by each reviewer. Please take careful note of the critical comments from Reviewers #1 and #3 on the figures and revise them accordingly for clarity to help non-specialist and specialist readers. Also, it is important that you seriously address the criticisms of reviewer #3 regarding communicating the implications of your study. We will also require a detailed list of your responses to the review comments and a description of the changes you have made in the manuscript.

**Reviewer #1 major comments**:

1. It seems unlikely that any of the Aphidini-associated Buchnera are able to produce PGN, given the lack of MurC, MurE and MurF. Rather, it seems like the PGN pathways in these endosymbionts are in the process of degradation – perhaps enabled by the loss of AmiD in the hosts. Have the authors checked whether the remaining genes are full length and not any pseudogenes?

Author response: For the genes included in Fig 3 (the gene presence/absence matrix), only genes predicted to be full-length coding sequences were included. We checked for pseudogenes following bacterial genome annotation using DFAST by removing coding sequences containing internal stop codons and/or frameshift mutations. Thus, none of the bacterial genes are predicted to be pseudogenes. We added a statement to clarify the purpose of this step in the methods section on line 633.

2. Related to this: do the authors have any evidence for PGN in these organisms? Bacterial cell shape, mass spec/PGN probes, or expression of Aphidini? Or is there any in the literature? Same question for the Macrosiphini associated Buchnera. Has PGN been directly detected in these? If so this should be mentioned and if not this should be pointed out too.

Author response: While several studies have demonstrated the presence of cell wall and peptidoglycan in multiple species of tribe Macrosiphini aphids (Hinde, 1971, *J. Insect. Physiol.* 17: 2035-2050; Houk et al., 1977, *Science*; Douglas et al., 2011, *Proc. R. Soc. B*), to the best of our knowledge, no data exists that indicates whether Aphidini *Buchnera* produce cell walls and/or PGN. As the reviewer suggests, recent comparative genomics-based approaches also hypothesized that Aphidini aphids (represented by *Schizaphis graminum* only) might not produce PGN at all (Atwal et al., *mBio*, 2021).

We acknowledge that the additional PGN pathway gene losses in Aphidini aphids described in our present analysis could suggest a nonfunctional pathway and the absence of cell wall in Buchnera from these aphid species, rather than functional linkage between the missing genes. To test this, we have performed additional experiments involving fluorescent labeling of the *Buchnera* cell wall with a D-Ala analog, ethynyl-D-Ala (EDA). These results demonstrate that *Buchnera* from Aphidini aphids are capable of building a cell wall and that the remaining PGN pathway genes of *Buchnera* are functional. Thus, the absence of *murCEF* and aphid *amiD* among virtually all Aphidini aphids is conspicuous and implies functional linkage. We incorporated these new results throughout the paper, including the title, abstract, and discussion.

3. It seems to me that these data show that ldcA is uncoupled from PGN synthesis in this system. The fact that Buchnera lack AmpG, AmpD and Mpl makes it difficult to imagine LdcA playing any role in this pathway and seems more likely to be carrying out an unrelated activity or perhaps interacting with a different host-associated bacterium (pathogens?).

Author response: We agree that aphid LdcA likely plays a role other than PGN recycling in *Buchnera*. Since the initial submission of the present study, we have since published a detailed biochemical investigation of *A. pisum* LdcA that demonstrates enhanced endopeptidase activity towards stem pentapeptides and stem peptide crosslinkages relative to *E. coli* LdcA (Smith *et al*., 2021, *mBio*). These substrates only occur external to *E. coli* cells, suggesting that aphid LdcA plays a role in degrading rather than recycling PGN, though the biological significance of these function is not yet understood. We have attempted to clarify the relationship of *ldcA* to the other PGN recycling pathway genes, including *ampG, ampD,* and *mpl*, while also including a reference to our newly published study of LdcA on lines 105-106, 336-339, and 393-396.

We also agree that LdcA activity may affect additional host-associated bacteria. We suggest that the retention of host *ldcA* in aphid species where *Buchhnera* lacks the PGN pathway could be related to the presence of co-obligate symbionts on lines 431-440, and suggest an alternative role on lines 440-444.

4. If AmiD is regulating bacterial levels, does this mean that the Aphidini are less well controlled than the Macrosiphini? Is there an alternative way that the macrosiphini are controlled or is there some other difference in the relationship that might have facilitated this?

Author response: In the discussion section, we suggest that because *Buchnera* experiences high levels of genetic drift that result in gene loss, a likely evolutionary trajectory is that the host loses its HTG(s) in response to their symbiont having lost the gene(s) that enable host HTGs to affect symbionts (lines 417-423 and 483-485). Thus, the absence of aphid *amiD* may suggest that the symbiont can no longer be controlled by whatever mechanism AmiD acts through because the symbiont no longer contains the target of AmiD. One could imagine, however, that the loss of the seemingly important ability to control *Buchnera* via AmiD could promote the evolution of alternative means of control, either by strengthening existing control measures or by evolving new ones. Thus, there may be significant differences in symbiont regulation between tribes Macrosiphini and Aphidini, which we allude to on lines 508-512.

5. Overall, make it clearer (ideally in the figures) which Aphids have possible additional endosymbionts because this could affect the rationale behind the presence of the PGN genes in the hosts.

Author response: To make it clear which species have additional symbionts, we altered Figs 2 and 3 by adding symbols adjacent to species names that designate either known co-obligate (†) or uncharacterized facultative symbionts (‡).

6. Did the authors check whether the genes being analysed were full length and not pseudogenes? If so, this should be mentioned.

Author response: See our response to reviewer #1 major comment 1.

**Reviewer #1 minor comments:**

1. Figure 1 legend, could add Macrosiphini and Aphidini in brackets after the end of the first sentence Aphidinae species to help readers.

Author response: We changed this sentence on lines 1092-1094 to read “Hypothetical metabolic reconstruction of *Buchnera* PGN metabolism with potential involvement of aphid HTGs in the Aphidinae subfamily (including tribes Macrosiphini and Aphidini).”

2. Fig 2 and 3 have been swapped – the legends don’t seem to match the figures as submitted. I believe Fig 3 is actually 2 and vice versa

Author response: We accidently uploaded the Fig 2 image as Fig 3 and vice versa; the legends are in the correct order.

3. Throughout, you can help readers who are not in the PGN field by being more explicit and adding more details. For example, in Fig 1 you could add FtsI in brackets next to PBP3. The authors use gene names in Fig 3 and protein names in Fig 1, which will be confusing to some readers. I would add more detail in brackets to nobody gets lost. For example, many of your readers may not be aware that mrdA is PBP2, mrdB is RodA.

Author response: We agree that for genes with distinct protein names, explicitly naming the corresponding protein in Figure 3 will improve clarity for non-expert readers. We have made the suggested change, placing PBP1B, PBP2, PBP3, and RodA in brackets besides the corresponding gene names *mrcB*, *mrdA*, *ftsI*, and *mrdB*. No other genes specifically named in these figures have such distinct protein names. For additional clarity, we added columns for “alternate gene names” and “protein names” to Table S8. Finally, we refer to the *mrcB* gene on lines 245-246; we added the protein name here to help readers follow along in Figure 1 and 3.

4. Similarly, in table S8 add common names PBP1/FtsI etc in addition to gene names. MrdB – rodA, mrdA – PBP2, mrcA, mrcB

Author response: See author response to reviewer #1 minor comment 3.

5. SEDS proteins (RodA/FtsW) should be included in Fig 1 if you want to show all glycosyltransferase activity

Author response: We agree and have added these proteins to Fig 1. We made additional minor modifications to the positions of arrows and gene names in the figure that we felt improved clarity.

6. I don’t have specific advice on how to fix this but I found Fig. 2 (called Fig. 3 – the one with gene gain/loss) very confusing to follow. I wonder whether there was any way the data could be presented more straightforwardly, perhaps by lining up the genes at the top of something. Once I figured everything out it was very comprehensive but I fear you may lose some readers with the complexity.

Author response: To Fig 2 more clear, we have taken the reviewer’s suggestion and added gene labels above the box previously labeled “species-specific gene gains/losses”. This change makes the final gene tally more easily read, as the reader no longer needs to look at the shape/color key, which is now only necessary to determine at which point in the tree a gene was gained or lost. We made additional minor modifications to the key in the figure that we felt improved clarity.

7. A specific note on this figure, the line to the right showing the superfamily Aphidoidea needs a line at end of Hormaphidinae to show that the others are from a different superfamily.

Author response: To better show the distinction between superfamilies in Fig 2, we changed the background color of the outgroup superfamilies and also moved the outgroup superfamily names to the right edge so that they’re no longer in the same column as subfamily names.

8. Fig 3 – you could help the reader by stating the superfamily along the top (even though they are all in the same). None aphid people will not be familiar with all of these so this will help readers move between Figs 2 and 3.

Author response: We agree and have made the suggested change to Fig 3, as well as additional minor modifications to the positions of lines and pathway names in the figure that we felt improved clarity.

9. Fig 3, colored circles are unclear. Are they all supposed to be part of a single pathway? There are only pink circles and one black one. Not sure why these genes were selected.

Author response: We included colored circles to indicate genes that participate in multiple pathways. We felt this information was significant because it shows that all of the cell wall synthesis genes retained by aphid symbionts are also implicated in cell division, revealing a possible functional link between PGN synthesis and cell division. We made changes to the final sentence of the Fig 3 legend that we feel improve clarity (lines 1125-1126).

10. line 293 – not sure this is really surprising, cell division can happen without PG eg chlamydia Ehrlichia. Could mention these.

Author response: We included a reference to recent comparative genomics studies that describe intracellular bacteria such as *Ehrlichia ruminantium* with similar gene repertoires to *Buchnera* from *C. viminalis*; lacking the PGN pathway but maintaining some cell division pathway genes (Otten et al., 2018, *Mol. Microbiol.*; Atwal et al., 2021, *mBio*). However, we are unaware of any scenario in which all cell division AND PGN genes are absent from bacterial cells capable of cell division, as is the case in *Buchnera* from *Stegophylla* sp. and *Pemphigus* sp. We altered lines 309-313 to reflect this important distinction.

**Reviewer #2 major comments:** Using comparative genomics, the authors revealed relationships between genes horizontally transferred from bacteria to aphids and the metabolism of peptidoglycan in their primary symbionts. The analysis is sound and thorough, with the obtained results interesting and important. I only have a few minor comments.

Lines 67-71: I understand what the authors mean. However, in many cases, Wolbachia, which is a main source of horizontally-acquired genes in host insects, is also categorized as a symbiont. I believe some modification is required.

Author response: We agree that Wolbachia can often be regarded as a symbiont, and that the bacteria from which aphid HTGs originate may have also been true symbionts. We have changed the sentence on lines 77-81 to, “…host genomes may instead harbor genes anciently acquired from bacteria other than the current symbiont(s) that have obtained roles in maintaining extant symbioses…”, removing the word “non-symbiont” and clarifying that the bacteria from which the HTG originates are no longer associated with the insect in question.

Lines 77-79: This appears exaggeration and inconsistent with the description at lines 215-218.

Author response: We believe that the exaggeration referred to is the apparent inconsistency of saying that *Buchnera* lack most PGN pathway genes on former lines 77-79 while saying that they harbor the most complete set of PGN pathway genes on former lines 215-218. We changed the latter sentence to read, “*Buchnera* from aphids of tribe Macrosiphini (subfamily Aphidinae) harbor the most complete set of PGN metabolism genes of all *Buchnera* species” (new lines 234-237), specifying that *A. pisum* *Buchnera* has the most genes of all *Buchnera* species, but still lacks the majority relative to *E. coli*. We made no changes to the first sentence, as this is in fact not an exaggeration – *E. coli* contains many more PGN pathway genes that are absent from the *Buchnera* genome. We hope that reviewer #2 understands and accepts our compromise to this comment.

Line 122, ~flagellar basal body (FBB) pathway: It would better to add a brief explanation here as to why this pathway was included in the analysis.

Author response: We changed the sentence on lines 132-135 in the introduction to more clearly introduce the idea that the cell division and FBB pathways overlap in function to some extent with cell wall metabolism. Later on (lines 304-306), we explain in greater detail how FBB assembly depends on a PGN hydrolase for insertion of secretion system through the cell wall.

Lines 271, 369, 543, Candidatus S. alterna: I prefer "Skilesia".

Author response: Except for the first instance, we have made the suggested changes and now refer to this symbiont as “*Skilesia*” throughout the manuscript.

Figures: It appears that figures 2 and 3 are oppositely placed.

Author response: See author response to reviewer #1 minor comment 2.

**Reviewer #3 major comments:**
I congratulate the authors for this interesting and thorough analysis, notably the sequencing of four additional aphid genomes. However, I had difficulties in appreciating the main implications, and I fear that these mainly descriptive results do not constitute a major advance for the subject, they are not conclusive on the function of the horizontally acquired genes other than what already known, nor is there experimental attempt at testing any arising hypothesis. As such, they may be suited for a more specialized audience than the large one of PloS Genetics.

Author response: The reviewer is correct that aphid HTGs have already been implicated in symbiont cell wall metabolism. However, while we hoped that our comparative genomics analysis would yield more conclusive evidence, what we attempted to determine was whether there is functional linkage between the three host HTGs themselves— that is, whether these genes function successively in a linear pathway, as mealybug HTGs do, or whether they have distinct and independent functions. We feel that our analysis provided ample data to refute the hypothesis that these enzymes are part of a single pathway. The uniform incidence of *rlpA* in the host genomes despite the presence/absence of a PGN pathway in symbionts implies that the primary function of *rlpA* is not to contribute to PGN metabolism; this was not known or even suggested prior to this work. As for *ldcA* and *amiD*, they differ enough in their incidence among aphid genomes that they, too, appear to be functionally independent of one another, although each appears related to symbiont PGN metabolism gene incidence.

In addressing reviewer #1’s comments regarding our lack of experimental evidence, we have since added results that directly test the conclusion that Aphidini tribe aphids are not be capable of synthesizing a cell wall, a hypothesis derived from our observation that Aphidini aphids lack *amiD* and their *Buchnera* lack 2-3 genes of *murF, murC,* and/or *murE*. Despite genetic evidence for the contrary, we detected cell walls in *Buchnera* from the Aphidini tribe member *R. maidis*. We have since incorporated these results throughout the manuscript, including the Abstract. We hope that reviewer #3 finds these additional results more conclusive.

Moreover, I found that the manuscript lacked in clarity and organization. Below some specific comments that may help improving it.

Figure 1 is very complete but also complex to look at. The authors may want to highlight the genes mentioned in the text (bold or red font for example).

Author response: We feel that the changes made while addressing reviewer #1 minor comments 3 and 4 have made Fig 1 significantly easier to follow alongside the gene and protein names used in the text, and hope that the reviewer will agree that this resolves any confusion.

My major concern if with Figure 2, which does not help understanding the results. There is no aphid tree in this figure, so it is impossible to follow the evolutionary events of loss and gain of PG genes. Figure 3 is not better, and I had a lot of trouble to go back and forth from it and the text.

Author response: We feel that the reviewer may have been confused by our accidental switch of Figs 2 and 3, resulting in mismatched legends and figure images. See author response to reviewer #1 minor comment 2. We hope the changes described there will also resolve the issues for reviewer #3.

The homologues were identified by simple BLAST searches, while more sensitive analysis (ex. HMM) may have been more suited, for example in the event of degenerated sequences or pseudogenization, a possibility not mentioned in the text.

Author response: The changes we made in response to reviewer #1 major comment 1 may resolve some of the concerns of reviewer #3 here. To address the other part of this comment, we felt that BLAST was a sufficient method for the identification of additional bacterial genes within insect genomes, especially those involved in a bacterial pathway that eukaryotic organisms typically lack altogether. However, the idea that additional HTGs exist in some aphid species is interesting and compelling— if *A. pisum* contains so many HTGs, other species could contain additional HTGs. Because this paper focused on whether a relationship exists between the known aphid HTGs and symbiont PGN metabolism, we did not consider the use of more sensitive tools to look for additional HTGs, but do not discount their potential existence.

All results described in pages 10-15 are not mentioned nor resumed in the abstract, and this is a pity. Even the title of the paragraph at line 208 does not make justice of all the work presented. Also, the hypothesis at the base of the analysis (that loss of PG remodeling enzymes in the aphid genomes may coincide with loss of PG synthesis in the symbiont, indicating functional linkage) seems trivial and may be better formulated.

Author response: We have rewritten most of the abstract and believe that it now includes results from former pages 10-15. We omitted some of our more specific findings from the abstract in order to avoid overstating conclusions lacking experimental evidence.

We agree that our hypothesis is an obvious one based on known gene functions in model species. But we maintain that it is non-trivial, especially in light of the experimental results we have added in the revision. Prior to our analyses, knowledge was limited by the lack of sequenced genomes. Also, before our own recent publication in mBio (Smith et al., 2021, *mBio*), no studies have attempted to test the idea that host HTGs might compensate for the absence of particular functions in symbiont PGN metabolism, though the original article describing the discovery of HTGs in aphids initially proposed this idea (Nikoh and Nakabachi, 2009, *BMC Biol.*; Nikoh *et al.*, 2010, *PLoS Genet.*). Even these studies stopped short of proposing any mechanistic hypotheses for how these genes might contribute to *Buchnera* proliferation*.* Because this hypothesis has not been overstated within the literature, it is valuable to begin our analyses with this simple question.

Line 330, put "Discussion" in bold.

Author response: No section headers are currently written in bold. For consistency, we have not incorporated the reviewer’s suggestion, but will be sure to follow the journal’s manuscript style upon acceptance.

In conclusion, this is an interesting -although largely descriptive- analysis which may deserve a better structured text and clearer figures to highlight the novelty and impact of the results for the large audience.

Author response: We feel that the addition of experimental data showing the existence of cell walls in Aphidini aphid species, made in response to reviewer #1 comment 2, has sufficiently raised the novelty and impact of our results to the level of the PLoS Genetics audience, and hope reviewer #3 would now agree.
