# Supplementary material for: Elucidation of host and symbiont contributions to peptidoglycan metabolism based on comparative genomics of eight aphid subfamilies and their Buchnera
Source: PLoS Genet. 2022 May 6;18(5):e1010195. doi: 10.1371/journal.pgen.1010195 (PMC9116674; doi:10.1371/journal.pgen.1010195)
Supplement: Attachment — Submitted filename: v3.4_review_comments_v2.docx [file pgen.1010195.s018.docx]

Editor comments:

The manuscript was fully evaluated at the editorial level and by independent peer reviewers. The reviewers appreciated the attention to an important topic but reviewer #1 identified some concerns with the lack of an appropriate negative control in Fig. 5 that we ask you address in a revised manuscript. In addition, reviewers #1 and #3 have made suggestions to improve the clarity of the text that we suggest you implement.

We therefore ask you to modify the manuscript according to the review recommendations. Your revisions should address the specific points made by each reviewer.

In addition we ask that you:
1) Provide a detailed list of your responses to the review comments and a description of the changes you have made in the manuscript.
2) Upload a Striking Image with a corresponding caption to accompany your manuscript if one is available (either a new image or an existing one from within your manuscript). If this image is judged to be suitable, it may be featured on our website. Images should ideally be high resolution, eye-catching, single panel square images. For examples, please browse our [archive](https://journals.plos.org/plosgenetics/volume). If your image is from someone other than yourself, please ensure that the artist has read and agreed to the terms and conditions of the Creative Commons Attribution License. Note: we cannot publish copyrighted images.

Reviewer comments

Reviewer #1: I am very happy to see the new EDA labelling data, this really strengthens the paper. However, I am concerned by a lack of appropriate negative control in Fig. 5. These probes are known to give high background labelling and whilst drug treatments are used to show specificity in Fig. 4 this uses a different labelling protocol in which bacteria are isolated from aphids. Therefore a suitable negative control is needed for Fig. 5 such as including PG targeting drugs during the last couple of hours of labelling or something similar.

Author response: The control experiment recommended by the reviewer would involve treating aphids with antibiotics such as ampicillin that target PGN synthases and then demonstrating a lack of incorporation of EDA. This strategy is not likely to work for the aphid-*Buchnera* system. It has been known for some time that ampicillin treatment does not reduce *Buchnera* titers in aphids, though it is effective at eliminating secondary symbionts like *Serratia* (Koga *et al*., (2007), *FEMS Microbiol. Ecol.*). While the exact reason has not been tested, ampicillin and other PGN-targeting antibiotics appear not to affect *Buchnera* in the same way as other bacteria.

We argue that a suitable negative control has already been provided for two main reasons. 1) A major source of high background fluorescence is non-specific binding/incorporation of the probe. The ELA probe tests for this because it should be incorporated into all proteins. There would be some cause for concern if the ELA probe had high fluorescence signal, but we show qualitatively and quantitatively that it does not. Additionally, we are administering these probes first to plants and, following transport through the plant vascular system, they are ingested by aphids*.* The fact that EDA somehow does make it to *Buchnera* in sufficient quantities for visualization, even after passing through both plant and aphid, is indicative of its specificity for PGN, which both plant and aphid lack. 2) The labeling protocol used for the experiments in Figures 4 and 5 was mostly the same, the only difference being that the bacteria were removed from the host following probe-feeding in the Figure 4 experiment while the bacteria were left intact within the host in Figure 5. In this light, the bacteria shown in Figure 4, which provides the proof-of-concept of PGN-labeling in *Buchnera*, were subjected to more overall sample processing than those in Figure 5. Thus, if the enzyme treatments in Figure 4 are considered trustworthy, then they should also convince readers of the data shown in Figure 5.

The figure legend for Fig. 4 does not describe which strain is being used.

Author response: We made several changes to the Figure 4 legend as well as the Figure 5 legend and methods section (lines 649-651, 719) to make clear which aphid lines were used for which experiments. We also added a label in Figure 4 and changed the color of the cartoon aphids to indicate the aphid strain used in these experiments (LSR1-c aphids are red, not green).

The use of different aphid strains between Figures 4 and 5 is derived from distinct goals for each experiment. In Figure 4 we used LSR1-c, the most commonly used *A. pisum* strain in our lab, to establish the proof-of-concept, while in Figure 5 we sought to determine whether different symbionts living in the same host background are labeled to different extents —the Austin strain is available both with and without a co-occuring *Serratia* symbiont, but comparable LSR1 strains are not.

The labelling in figures 4 and 5 is unclear. Labelling microscopy panels as "AF488" or "cy5" is not helpful - the reader needs to know what is being labelled. So "EDA" or "PG label" is much more informative.

Author response: We agree that the labeling could be improved. We changed the microscopy image labels in both Figures 4 and 5 and in their respective legends to indicate the target of the probe rather than the probe itself.

Reviewer #2: I believe this revised version is suitable for publication in PLOS Genetics.

Reviewer #3: The authors have answered my comments and the manuscript is now much clearer. They have also addded critical additional data that strengthens the impact of the work.

I still find figure 2 hard to follow but I understand you have done your best. I remain very skeptical on how species lacking murCEF can still produce PGN.

Author response: We agree that this result is surprising. We attempted to offer a plausible explanation in the Discussion section (lines 481-495). In short, we hypothesize that multifunctional enzymes may be involved in cell wall synthesis in *Buchnera* species lacking *murC*, *E*, and/or *F*.

A few minor comments:
Abstract line 37: "The loss of amiD and ldcA HGTs coincides with symbiont PGN metabolism genes". This sentence is not very clear, i suggest to replace by "The loss of amiD and ldcA coincides with that of symbiont PGN metabolism genes".

Author response: We acknowledge that this sentence is unclear and have changed this sentence to read “the loss of aphid amiD and ldcA coincides with the loss of symbiont PGN metabolism genes”. However, we made additional changes to this sentence described in the response to the next comment (lines 38-39).

Line 41: I was confused by you putting forward a hypothesis and then discarding it. Maybe add "The coincident loss of host amiD and symbiont murCEF in tribe Aphidini, in contrast to tribe Macrosiphini, may suggest...."

Author response: We agree that this was confusing and have reworded this sentence to address the reviewer’s concern. To make this clearer, we made an alternative change in lieu of the reviewer’s suggestion which we hope will address the root of the reviewer’s concern (see lines 37-43). We now list the two competing hypotheses that our initial manuscript failed to adequately test, but that are addressed by the additional experiments shown in Figures 4 and 5.

Line 61 "involvement in the synthesis of the symbiont cell wall"

Author response: We changed this phrase to “involvement in symbiont cell wall synthesis” (line 62).

Line 143: I would add: "In contrast with this hypothesis, we provide experimental evidence for ...."

Author response: We have made the suggested change (line 144).

Additional changes made:

The l and d configurations of some chemicals were not formatted correctly as small caps and were thus changed (lines 655, 657-658).

We added a triangle in the background of Figure 5 intended to make clear that the images in panel B are derived from a particular column of images in panel A.
